# Improved urban runoff prediction using high-resolution land-use, imperviousness, and stormwater infrastructure data applied to a process-based ecohydrological model

**DOI:** 10.1371/journal.pwat.0000155

**Published:** 2023-11-20

**Authors:** Jonathan Halama, Robert McKane, Bradley Barnhart, Paul Pettus, Allen Brookes, Kevin Djang, Vivian Phan, Sonali Chokshi, James Graham

**Affiliations:** 1 U.S. Environmental Protection Agency, Corvallis, OR, United States of America,; 2 NCASI, Corvallis, OR, United States of America,; 3 Inoventures Inc., Corvallis, OR, United States of America,; 4 Cal Poly Humboldt, Arcata, CA, United States of America

## Abstract

Modeling large-scale hydrological impacts brought about by site-level green and gray stormwater remediation actions is difficult because urbanized areas are extremely complex dynamic landscapes that include engineered features that, by design, expedite urban runoff to streams, creeks, and other water bodies to reduce urban flooding during storm events. Many urban communities use heavily engineered gray infrastructure to achieve that goal, along with more recent additions of green infrastructure such as rain gardens, bioswales, and riparian corridors. Therefore, successfully characterizing those design details and associated management practices, interactions, and impacts requires a detailed understanding of how fine and course-scale hydrologic processes and routing are altered and managed in urban watersheds. To enhance hydrologic modeling capabilities of urban watersheds, we implemented a number of improvements to an existing ecohydrology model called VELMA—Visualizing Ecosystem Land Management Assessments—including the addition of spatially explicit engineered features that impact urban hydrology (e.g., impervious surfaces, curbed roadways, stormwater routing) and refinement to the computational representations of evapotranspiration by adding impervious surface evaporation. We demonstrate improved capabilities for modeling within complex urbanized watersheds by simulating stream runoff within the Longfellow Creek watershed, City of Seattle, Washington (WA), United States (US) with and without these added urban watershed characteristics. The results demonstrate that the newly improved VELMA model allows for more accurate modeling of hydrology within urban watersheds. Being a fate and transport ecohydrology model, the improved hydrologic flow enhances VELMA’s current capacity for modeling nutrient, contaminant, and thermal loadings.

## 1. Introduction

Urban watersheds are dynamic, complex environments that contain a mixture of engineered and natural attributes, and it is therefore difficult to monitor, model, and fully represent small-scale attributes that collectively impact watershed-scale runoff. The reduction of stormwater runoff and contaminants using a variety of green and gray stormwater infrastructure has been studied extensively at the site level under a variety of conditions [1–5]. However, the ability to accurately model watershed-scale responses to site-level attributes has been mainly limited to either natural process simulations or engineered infrastructure simulations, resulting in neither a hydrograph nor pollutograph fully representing complex urban watersheds.

This lack of natural process simulations and engineered infrastructure represented together has left a gap in model-based representations of urban runoff that describe and predict the interacting effects of natural and engineered processes across multiple watershed scales. For example, at watershed scales the transport of water through landscapes is determined by several key surface and soil characteristics. Within a natural landscape, precipitation falls upon the terrain and vegetation. Local details will influence percentages, but in general ~10% of the water will transport through the watershed as runoff, ~50% will infiltrate into the shallow and deep soils, while as much as 40% will undergo evapotranspiration [6].

In contrast, semi-urban to urban systems are developed landscapes that consist of a nonnatural state of surface perviousness (e.g., concrete, asphalt, compacted soils) and contain engineered water routing systems, including but not limited to municipal separate storm sewer system (MS4) and combined sewer system (CSS) piping, detention ponds, culverts, vaults, urban wetlands). The foremost goal of these systems is to rapidly move water out of the urban areas to avoid urban flooding. Within urbanized areas approximately 55% of precipitation flows along infrastructure that routes, channelizes, and consolidates water within stormwater systems [6]. Water traversing along any exposed surface will either evaporate into the atmosphere or be routed by infrastructure (e.g., blacktop surfaces, street curbs, roadway drainage ditch), which in total can account for 85% of the watersheds total water budget (runoff plus evaporation) [6]. This water either reaches a more natural substrate (e.g., lawns, urban grass fields, open dirt areas), or is routed within the stormwater infrastructure to urban streams or directly into large water bodies (e.g., sounds, large river systems, ocean). As landscapes transform from natural to more urban conditions, less water is transported through ground infiltration (shallow and deep) and evapotranspiration, and more water is transported as surface runoff [6]. During major rain events this can overwhelm some stormwater infrastructure, leading to excess surface water and local to widespread flooding, overwhelming the CSS resulting in combined sewer overflow (CSO) events [7]. In addition, the reduction of natural processes in favor of engineered-urban conditions can reduce other ecosystem service benefits such as bioremediation, carbon sequestration, and fresh water for wildlife and fish habitats [8, 9].

The inclusion of green infrastructure (GI) and other forms of low impact development (LID) in urban landscapes has been demonstrated as an effective approach for both reducing peak urban stormwater flows and mitigating urban contaminants [1, 4, 5, 10–12]. However, GI is often not uniformly effective in both time and space. The efficiency of GI for reducing runoff and contaminants depends on the local surrounding environment and installed infrastructure’s ability to route distal upstream surface and subsurface runoff water and contaminants through GI treatments. In a paired urban landscape study, the addition of GI through a two-phase infrastructure project demonstrated stormwater peaks were reduced by 33% with a 40% total run-off reduction [13]. Similar results were observed in a neighborhood study where stormwater control measures (SCMs) in the form of curb-and-gutter stormwater conveyances and one vegetated swale were installed. Surface runoff for SCM-treated catchments went to zero for precipitation events under 6 mm, though flow reduction was not detected for the swale [14]. Conversely, as precipitation magnitudes exceeded 20 mm, the green and gray catchment became comparable, leading the authors to the conclusion that both placement and redundant “treatment trains” were likely needed to reach reduction goals [14]. A similar finding involving green roof modeling in the City of Seattle, WA, US revealed there is likely an upper limit as area of GI placement increases within a watershed area; meaning additional roof GI was effective for reducing peak storm flow within the urban streams, but only up to a theorized limit [5]. Methods for remediating urban impacts are limited with GI being an approach with multiple benefits but should be accounted for when modeling the hydrology of urban landscapes [15].

Semi-distributed modeling frameworks such as Storm Water Management Model (SWMM) and Soil & Water Assessment Tool (SWAT) are specifically designed for simulating the fate and transport of water, nutrients, and contaminants through urban watersheds at relatively low spatial resolution [16, 17]. When natural and engineered features affecting runoff coexist within specified hydrologic units, important process-level controls can be confounded across scales within the watershed boundary. Alternatively, fine scale modeling frameworks allow users to construct high-resolution simulations that capture the detail of urban landscapes (e.g., roads, parking lots, curbed streets, roof tops, stormwater drains, outfall locations). This also allows for explicit placement of gray and green infrastructure or other management actions that cannot be resolved by semi-distributed models [5, 12].

Here we developed such a high-spatial resolution framework by leveraging the Visualizing Ecosystem Land Management Assessment (VELMA) model, which has been demonstrated to suitably simulate hydrologic and nutrient transport mechanisms within natural (non-urban) and urbanized landscapes [5, 18–22]. VELMA is a spatially distributed (i.e., grid-based) ecohydrological model that simulates land surface hydrology and terrestrial biogeochemistry to characterize the integrated responses of vegetation, soil, and water resources to multiple interacting stressors and watershed management actions across a wide range of spatial and temporal scales–plots to regional basins, and days to decades [21, 22]. The novelty to this ecohydrology modeling approach is leveraging VELMA’s ability to simulate dynamic vegetation growth and landscape disturbance along with dynamic fate and transport of water and nutrients upon the surface (including evapotranspiration) presented here. For this study, we enhanced VELMA to improve daily and multi-year urban watershed runoff predictions in response to combined effects of high-resolution land-use, imperviousness, and stormwater infrastructure data. An overarching purpose of these hydrological improvements is to prepare VELMA for subsequent work to improve fate and transport predictions of urban stormwater contaminants and, ultimately, to inform GI best management practices for improving stream and estuarine water quality and habitat conditions.

Toward these goals, we developed VELMA to improve the model’s representation of urban hydrologic processes in four important ways: 1) by adding an explicitly modeled surface layer supporting 2D (cell-to-cell) lateral transport of water and chemicals that allows specification of cell-specific surface layer imperviousness (0–100%) to control partitioning of lateral and vertical (infiltration) transfers of surface layer water and chemicals; 2) added capability to route water along curbed streets, including transverse transfers across normal gravitational flow paths; 3) added capacity to transfer water through a piped network; and 4) enhanced calculation of spatially explicit surface evaporation to represent impervious surface evaporation.

We demonstrate these urban advancements of VELMA through a series of simulations of a densely urbanized watershed, whereby each enhancement is removed from a calibrated simulation set up using available high-resolution spatial and temporal data. This demonstration illustrates how changes in model representation, not parameter calibration, influence simulated runoff performance compared to observed data. The intent of these simulations is to demonstrate how each improvement is directly associated with VELMA’s capacity to accurately represent the influence of complex urban infrastructure on runoff and provide process-level insight useful for informing community stormwater management goals important to community, state, and federal partners.

## 2. Methods

### 2.1 Site description

**Fig 1.**
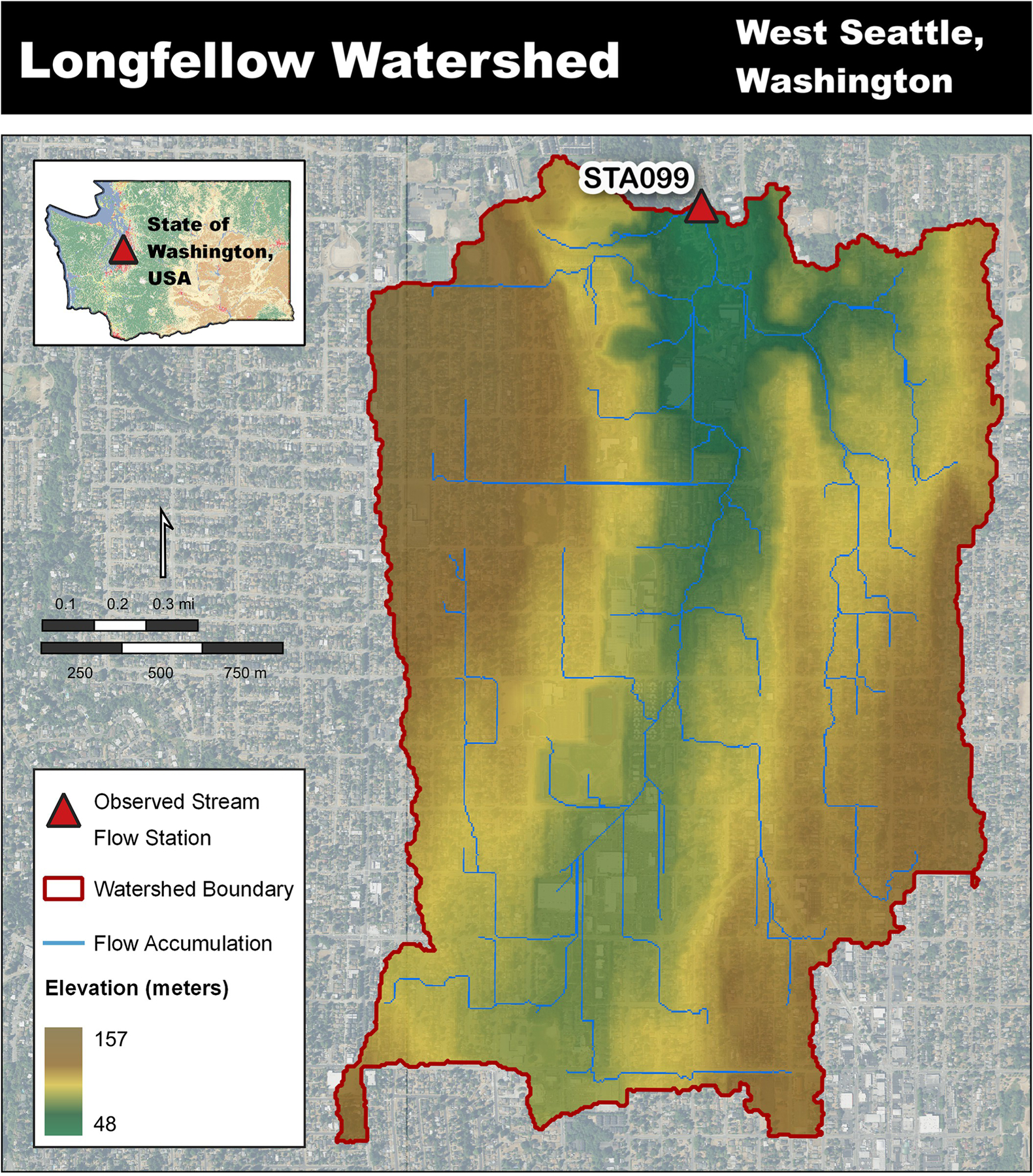
Longfellow Creek watershed site overview to King County observed stream flow station STA099 [23, 24].

The Longfellow Creek watershed encompasses a major portion of Seattle Council District 1 (portions of West Seattle, Delridge, and White Center neighborhoods) City of Seattle WA, US (Fig 1).

The watershed contains all the hydrologic complexity that exists in a typical urban watershed, including both separated sanitary sewer and stormwater drainage systems as well as a combined stormwater system and several water holding features, for example, stormwater vaults, detention ponds, and small lakes. This urbanized landscape also includes considerable areas of managed grass and forest cover types within lawns, boulevards, riparian zones, and a golf course. Assessment of roads, buildings, stormwater systems, and green stormwater infrastructure (GSI) in geographic information system (GIS) revealed a 5-meter (5-m) grid is a sufficient scale for modeling urban hydrology. The 5-m resolution sufficiently captures the street widths within neighborhoods and commercial zones, represents roof top areas within residential neighborhoods, and allowed sufficient representation of stormwater inlet placements.

Longfellow Creek itself runs south to north and has two observed flow stations (STA098a and STA099) managed by King County Hydrologic Information Center. Continuous daily stream flow data were available for the years 2019 through 2022 at station STA099 (47.54144°N, −122.36285°W), which allowed for testing of model hydrologic performance, yet did constrain this work to that timeframe [25]. With station STA099 defining the pour point the delineated area was 4.63 km^2^ or 1144 acres. The delineated landscape is represented by four coverages: grass (50.0%), trees (26.0%), buildings (17.1%), and roads (6.5%). Each building and road cell have a corresponding 100% impervious value making the total imperviousness 23.6% of the delineated area.

The Seattle Public Utility (SPU) stormwater system is represented in GIS with each drain input, lateral pipe, mainline pipe, and outflow location labeled through a numbering arrangement that allowed for the reliable and testable development of a stormwater representation within the VELMA model [26–28]. The 1-meter resolution land classification and impervious surface data was available from the City of Seattle, WA, previously used by Barnhart et al. [5]. Here, tree coverage was refined using the latest City of Seattle Light Detection and Ranging (LiDAR) derived tree canopy coverage [28].

### 2.2 VELMA model, framework, and terminology

**Fig 2.**
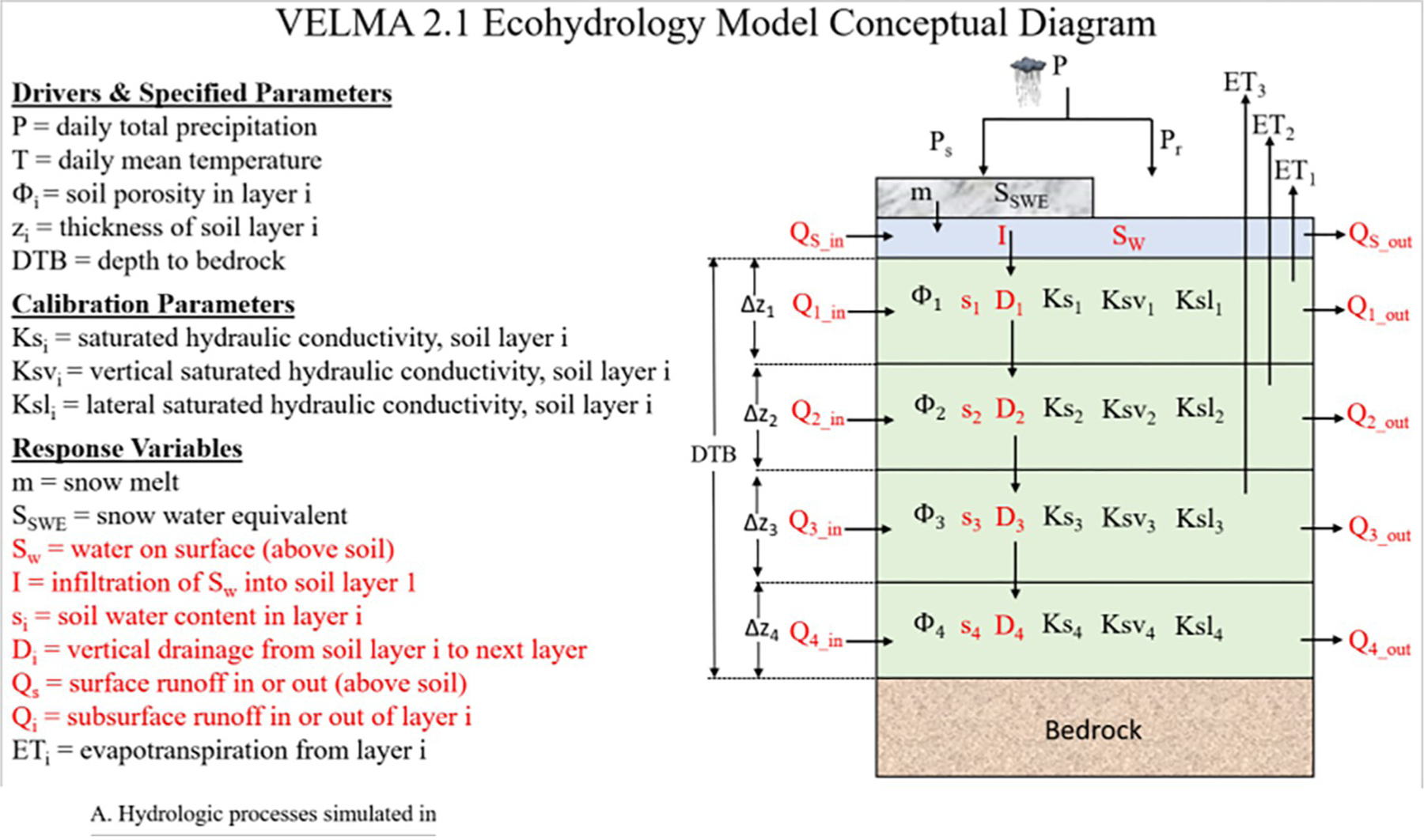
VELMA surface and four soil layer conceptual diagram.

VELMA is a spatially explicit ecohydrological model that includes multiple submodels representing integrated environmental components (hydrology and terrestrial biogeochemistry) to simulate the impacts of various stressors on dynamic responses of vegetation, soil, and water [21, 22]. VELMA parameters and watershed-scale simulation setup details are described in the 2014 VELMA 2.0 user manual [21]. Here we highlight key aspects of the model that were leveraged or enhanced to adapt VELMA for modeling urban watersheds. VELMA’s spatially explicit, gridded framework consists of a 2D surface layer used to model surface runoff on impervious to semi-pervious surfaces with four vertically delineated subsurface layers that allow water infiltration (Fig 2). The four soil layers represent vadose zone soils with user defined layer thicknesses. VELMA does not model water within deep (aquifer) layers. Listed in the following table are the soil parameters with the most influence on urban watershed modeling advancements (Table 1).

#### 2.2.1. Surface runoff model components.

We applied VELMA to the Longfellow Creek watershed using a 5-m grid to simulate effects of fine-scale infrastructure features on urban runoff. Each 5-m cell location was assigned a land cover/vegetation type based on City of Seattle high-resolution GIS data [5, 28]. Watershed vegetation cover types included trees, grass, and impervious surfaces such as roads, parking lots, and roofs. Plant biomass exists on all cells within the model, even if biomass is very low such as moss and small weeds. Growth on impervious surfaces occurs and if left undisturbed will reach unrealistically high levels. Therefore, all biomass growing on impervious surfaces were regularly suppressed through a biomass harvest/removal disturbance routine designed to maintain near bare surface conditions. This setup also left open the possibility of conducting future green roof simulations like the VELMA urban green roof applications described by Barnhart et al. [5].

#### 2.2.2. Subsurface runoff model components.

Soil layers 1 through 4 represent the four subsurface soil matrix layers. To simulate subsurface hydrology, each soil layer has a set of parameters used to mimic the movement of water through the soil matrix (Table 1). These parameters should all be considered and possibly calibrated when shifting a VELMA calibration from a nonurban to urban application. This was accomplished for Longfellow Creek watershed.

#### 2.2.3. VELMA ecohydrology model.

The VELMA 2.0 model was designed to simulate natural watershed hydrology processes through two core sub-models, “*Water-Balance*” and “*Plant-Soil-Model*”. *Water-Balance* tracks the state of water volume in five “spatial pools” representing the surface to near surface terrain [21]. The first (top) pool represents the 2D surface layer, while the landscape’s soil matrix is comprised of the four subsurface 3D voxels (volume cells). The cell (grid) size of these pools can be set to essentially any resolution that meets a model user’s objectives and for which spatial data are available–most commonly 10-m and 30-m grids due to ease of access to publicly available landcover, soils, climate, and other spatial data at these scales. Due to the detail required to explicitly represent roads impact on hydrology within the Longfellow Creek watershed, the simulations presented here used a 5-m resolution.

At the start of each VELMA simulation day, a driver file adds precipitation (mm) to the surface layer of all receiving grid cells. For every cell within the simulated watershed, the daily amount of water transferred vertically from the surface to the uppermost (layer-1) soil layer depends upon the soil’s unsaturated storage capacity (Fig 2). If the uppermost soil layer is saturated, water is transferred laterally along the surface layer to downslope cell neighbors according to elevation difference (i.e., per calculated gravitational flow paths, except when street curbs redirect water transversely across natural flow paths). Within the soil, water infiltrates vertically, layer to layer within a cell’s 4-layer soil column, again dependent on the receiving cell’s unsaturated storage capacity. Otherwise, water exceeding saturation capacity moves downslope laterally to cell neighbors. VELMA calculates daily changes in the degree of saturation within every soil layer within each watershed cell based on soil porosity, bulk density, layer thickness, total soil column depth, and calibrated parameters (Fig 2). Finally, modeled surface and subsurface flow paths are used to categorize cells as “channel” or “non-channel”, such that daily surface and subsurface water transfers from non-channel to stream channel cells are reported as watershed runoff for that day.

Evapotranspiration in VELMA is simulated via its *Plant-Soil-Model* (PSM) sub-model. PSM manages vegetation growth including the uptake of water and nutrients from the soil matrix, while the *Water-Balance* sub-model is responsible for routing and tracking water [21]. Briefly, plant roots take up water as a function of several parameters (amount of available water between field capacity and wilting point, plant root biomass, and others). Evapotranspiration (ET) is simulated as soil water taken up and transpired by plants to the atmosphere (“T” in ET), plus surface water evaporated (“E”) to the atmosphere. For the US Pacific Northwest region ET is approximately 20–30% of the total water balance [29, 30].

Urban watersheds contain non-natural components that impact hydrology as well. These include stormwater systems such as drains, piping, and outflows as well as other semi-natural features such as urban lakes and detention ponds. In addition, urban watersheds contain a higher percent of impervious surfaces that prevent water penetration into the soil matrix, route water, and shift evaporation rates. Modeling of these features found in urban environments were built into VELMA and are described below.

#### 2.2.4. Enhanced framework for urban modeling applications.

VELMA has reliably estimated hydrological and biogeochemical responses to change in natural systems but has lacked important stormwater infrastructure and other features to accurately simulate flashier patterns of runoff observed in urban watersheds. To improve the applicability of VELMA for urban watersheds, we enhanced the model in four ways:

- *Enhancement 1*: Inclusion of partial to fully impervious surface.
- *Enhancement* 2: Inclusion of piped network (e.g., MS4 and CSS stormwater systems).
- *Enhancement* 3: Inclusion of curbed streets.
- *Enhancement* 4: Impervious surface evaporation

VELMA’s *Water-Balance* sub-model carries out the model’s explicit cell-to-cell transfer of water, calculates the daily flow volumes, and summarizes the daily flow results as annual statistics. This core section of the model’s functionality was left intact to ensure the model still functioned well within natural landscapes. The water fate and transport enhancements to allow urban watershed applications was carried out through the addition of an impervious map and through the inclusion of a stormwater water disturbance feature, both described below.

##### VELMA Enhancement 1: Impervious surface.

**Fig 3.**
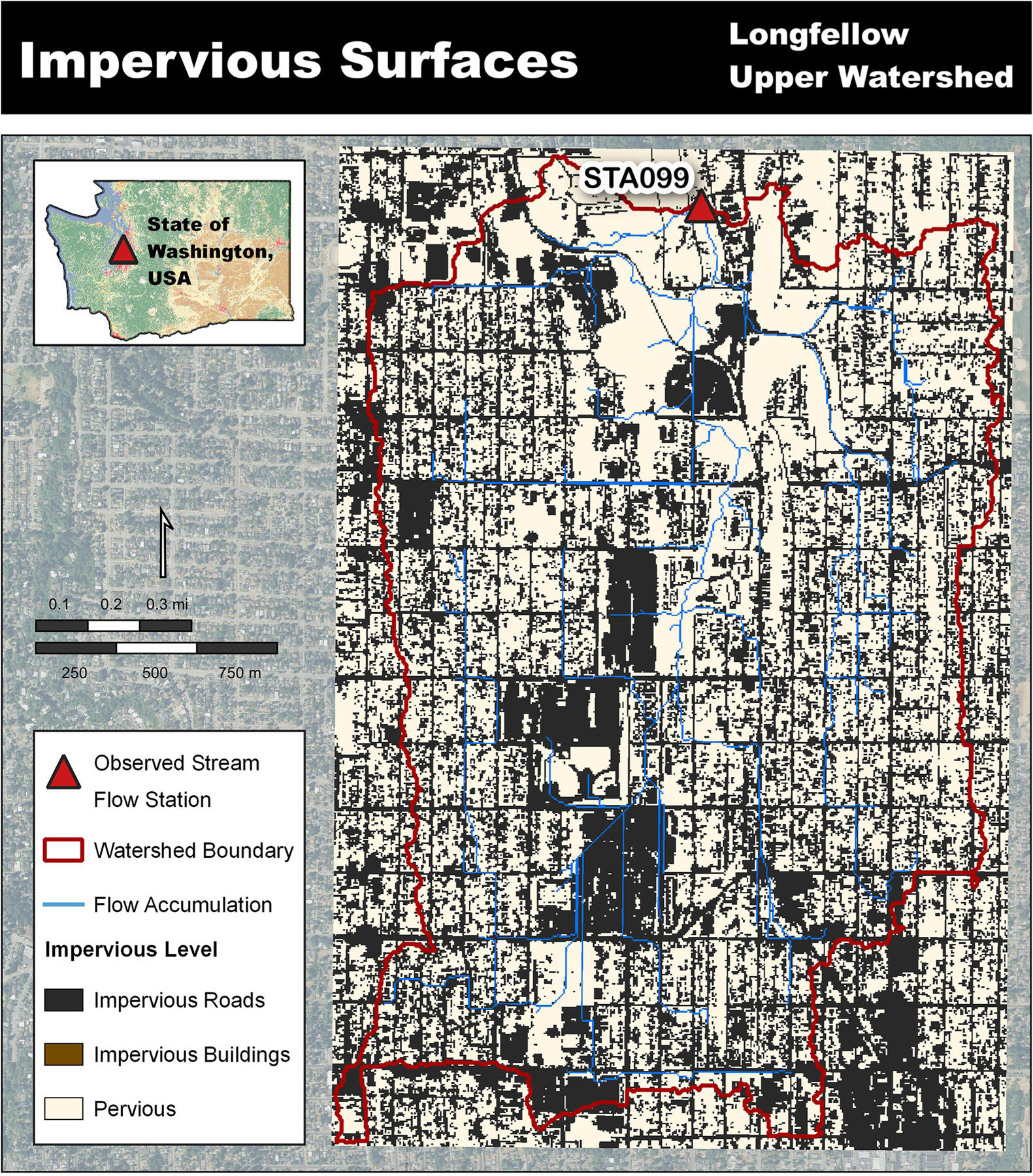
VELMA’s impervious surface representation corresponding to building roofs and roadways [23, 24].

The VELMA Model possesses a surface representation for modeling precipitation, snow, air temperature, and surface organic material such as detritus, though a surface imperviousness representation did not exist. The surface layer was enhanced to enable users to specify surface layer perviousness per grid cell via a map, whereby a cell’s perviousness can be assigned as a floating-point map value between 1.0 (100% pervious) to 0.0 (100% impervious) (Fig 3).

This provided a mechanism to control water infiltration from the surface layer into the first soil layer. Any water not allowed to infiltrate remains on the surface and becomes excess water that will transfer to neighboring downslope cells.

An urban watershed setup can include an imperviousness surface with values ranging between 0.0 and 1.0. For the Longfellow Creek watershed setup all road and building cells were assigned a value of 0.0 (fully impervious) due to the 5-m resolution allowing homogeneous representation of those coverage types (Fig 3).

##### VELMA Enhancement 2: Curbed streets.

**Fig 4.**
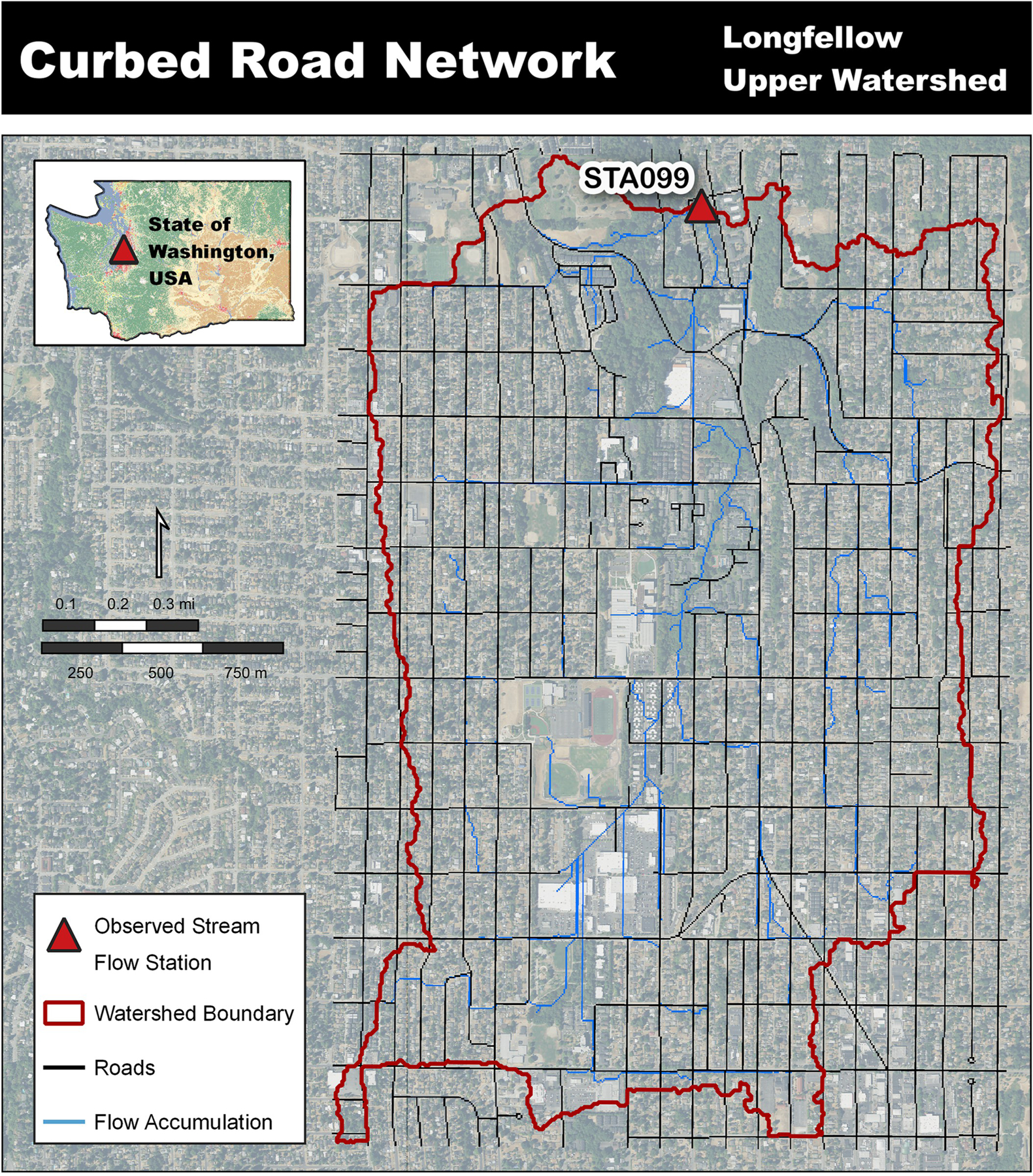
Stormwater curbed street inclusion through use of the water-disturbance routine [23, 24].

Street systems within an urban landscape can include curbs or drainage ditches that route water along roadways to curb stormwater drain inlets or catchment basins. These engineered systems shift water away from natural (gravitational) flow pathways to instead transfer water either 1) unnaturally elsewhere within the watershed delineation (e.g., stream, detention pond, stormwater overflow vaults) shifting the hydrograph peak and timing, or 2) diverting water outside of the watershed delineation. When externally diverted water enters the stream system below the monitoring location or is routed outside the watershed delineation, that water is removed from the originating watershed’s hydrograph. To accurately represent water routing through both the natural landscape and engineered stormwater systems the water must be explicitly routed along the surface and through the watersheds infrastructure but executed within the constraints of the model’s framework. Curbed street network was representation within the Longfellow Creek watershed setup (Fig 4).

Within VELMA, water movement along curbs and within stormwater systems is processed by a cell-to-cell water transfer disturbance. If a cell-to-cell curb disturbance is in place, and water is present at that cell’s surface layer, the water transfer disturbance action will transfer water from cell-A to cell-B based on either the water percentage or water volume controlled by the disturbance action. The cell-to-cell transfer and water percentage or volume is all provided by the model user during the “*Water-Disturbance*” action setup.

The details of these water transfers are closely intertwined with stormwater system water transfers. The curb-to-curb, curb-to-inlet, and stormwater inlet-to-outlet are typically represented in one disturbance file and built into the same *Water-Disturbance*, therefore further details on both are described in the following section.

##### VELMA Enhancement 3: Piped network.

Stormwater piping systems greatly influence how water routes through and out of a watershed. To enhance VELMA urban stormwater modeling capabilities, high-resolution GIS data have been added to represent spatially explicit stormwater inlets, pipe network, and outflows [26–28]. Within VELMA’s framework, stormwater movement is explicit regarding the starting inlet and ending outflow locations. VELMA is limited to only controlling the volume of water transferred within the pipe at a daily timestep, unlike SWMM that explicitly includes pipe diameter and length, stormwater vaults, and simulates water movement on an hourly timestep [31]. Within a VELMA simulation timestep, if a cell has been designated as a curb cell or stormwater inlet cell, any available surface water will be transferred from said cell to its corresponding transfer cell. This transfer of water does not explicitly account for stormwater carrying capacity due to limitations in pipe diameter or length that could result in the stormwater system being overwhelmed within less than a oneday timestep.

**Fig 5.**
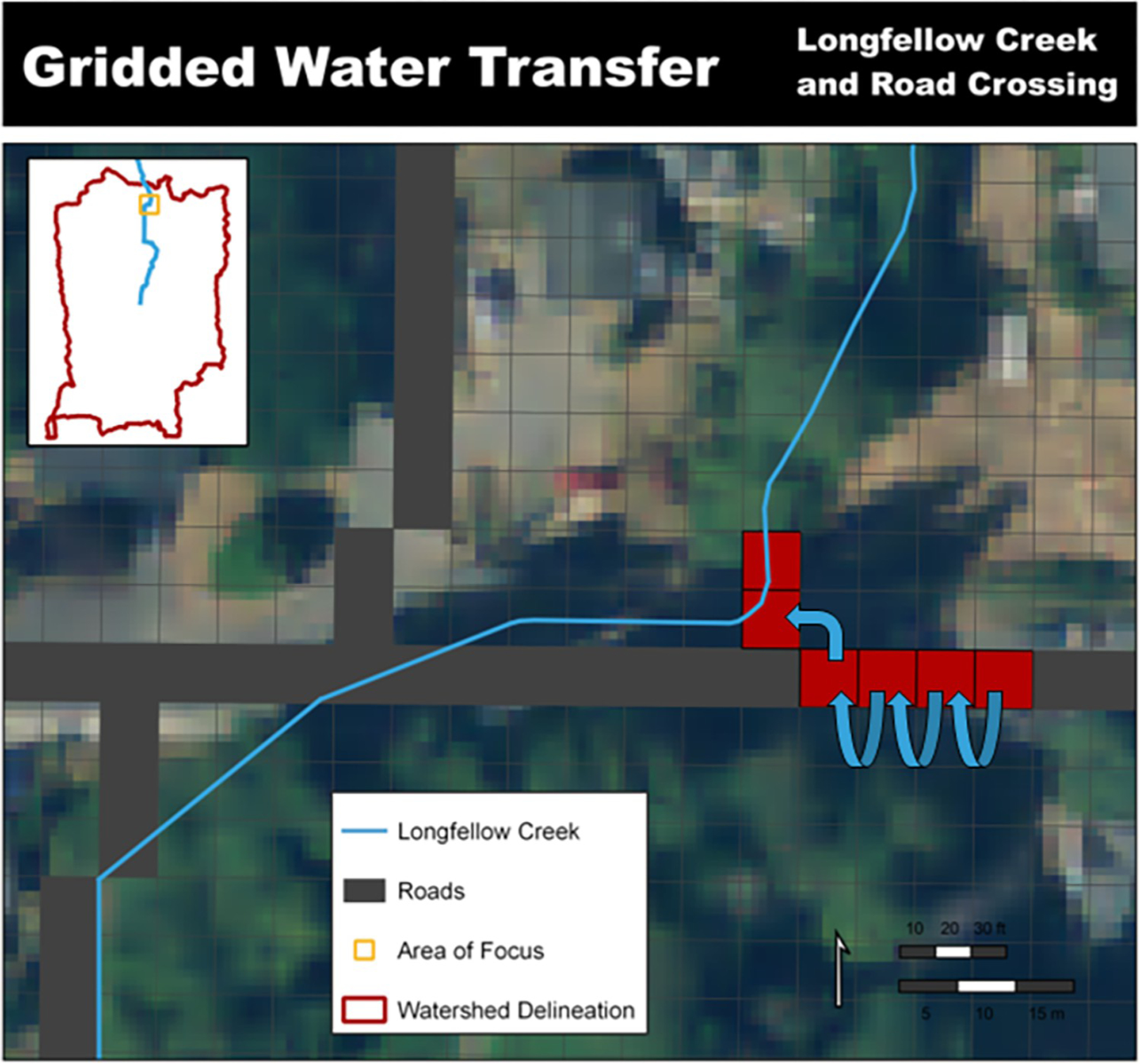
Water-disturbance explicit water transfer examples. VELMA is a fully gridded spatial framework, but for simplicity only small specific set of cells are represented in this map image overlay to convey the mechanism of curb and stormwater transfer actions occuring within VELMA during a simulation. Values 1 through 4 in this map image correspond to the first column of values in Table 2 [23].

These urban water controlling features are provided to the simulation through a CSV data file dictating water and chemical transfers from cell-A to cell-B water due to engineered developments (e.g., stormwater piping, culverts, curbs, piped roof drainage) (Fig 5 and Table 2). Data detailed in this water disturbance CSV file dictates the i-index (1D unique value within a 2D grid) location of where water moves from, volume or percent of water transferred, and the cell location[s] water is transferred.

A brief representation of transfers is presented with corresponding transfers described (Fig 5 and Table 2). Transfer numbers ‘1’ and ‘2’ denote curb-to-curb transfers occurring along the roadway to number ‘3’ Curb-to-Catchment transfer followed by the number ‘4’ Catchment-to-Outflow transfer.

For all water disturbances prescribed in a disturbance table, water transfers can only occur when water is present on the surface. For curb-to-curb water disturbance actions, these water transfers emulate the lateral movement of water downslope along the surface of a curbed street, usually to a stormwater inlet or drainage area.

**Fig 6.**
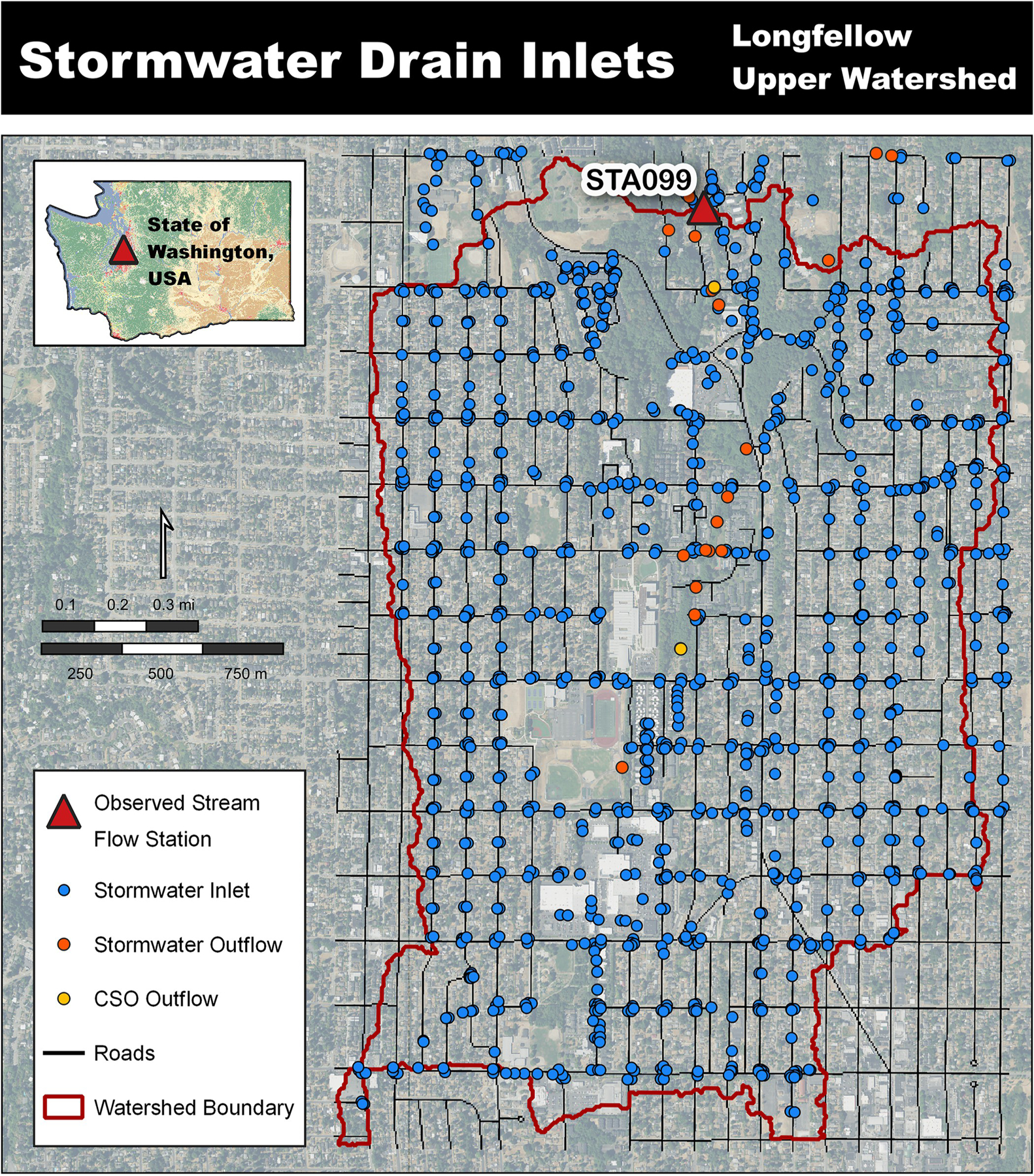
Stormwater catchment to outfall inclusion through use of the water-disturbance routine [23, 24].

Urban watersheds often possess stormwater systems that route water in directions contrary to natural flow paths (Fig 6). The VELMA disturbance module called “*WaterDrainDisturbanceModel*” was developed to quantitatively describe spatial and temporal transfers associated with this new water routing mechanism. This disturbance routine allows for all or a portion of any surface water at an assigned surface cell to be transferred to any other assigned surface cell.

Featured here are three uses of the “*WaterDrainDisturbanceModel*” to realistically represent the movement of water through the upper delineation of Longfellow Creek watershed to STA099.

- Curb-to-curb disturbance: this disturbance moves surface water from one road cell to the next in a sequence representing lateral movement of water downhill along a curbed street.
- Stormwater inlet-to-outlet disturbance: this disturbance transfers water from multiple street stormwater inlets to the stormwater outlet designated by the stormwater GIS pipe data.
- Roof Drainage inlet-to-outlet disturbance: this disturbance transfers all water from roofs that have corresponding “lateral drainage” pipes out of the delineation.

Within these piped networks, the transfer of water for three different cases can be represented, where: 1) a singular input location transfers water to another singular outflow location (e.g., culverts, or tunneled streams), 2) multiple input locations transfer water to a singular outflow location (e.g., stormwater system inlets routing to one outflow location, inputs or drainage fields routed to a detention pond), and lastly 3) multiple input locations transfer water to multiple outflow locations (e.g., small to large stormwater system with multiple outflow locations, segmented stormwater system where water outflows into a stream, but then downstream water is piped again through a tunneled stream). All these installations alter the timing of water transporting through the landscape, whether through urban impervious areas or natural landscapes. The curb-to-curb action is crucial for accurately modeling water movement on impervious roadways; plus is vital for simulating urban contaminant transfers along the same roadways.

If the final curb-to-curb cell transfer in the sequence is to a drainage field or a bioswale, the water transferred will work vertically downward into the soil matrix. If the final cell transfer is curb-to-inlet, the transferred water will enter an explicit location within the stormwater system and transfer directly to an explicit outflow location, as designated by the stormwater GIS data.

##### VELMA Enhancement 4: Surface evaporation.

Direct surface evaporation can be a considerable portion of a watershed’s total water budget. VELMA 2.0 modeled ET as the sum of evaporation of water on land surfaces plus plant transpiration, i.e., the process by which plants utilize soil water and evaporate it through pores in leaf surfaces. However, while VELMA 2.0 explicitly (mechanistically) simulated transpiration, evaporative losses from land surfaces are implicitly modeled based on watershed-scale water balance calculations. Owing to the importance of urban impervious surfaces evaporation, VELMA now explicitly models surface evaporative loss independent of plant evapotranspiration.

As landscapes transform from natural to urbanized, there is a large shift from vegetated soils to soils covered by impervious surfaces (e.g., concrete, asphalt, brick). This landscape change results in a new environment characteristic that possesses the process of direct evaporation from the surface to the atmosphere. To represent this new process the *SurfaceEvaporation* model was created and added to VELMA as a map-based component of a simulation. When *SurfaceEvaporation* is included in a simulation run, surface evaporation (mm/d) is calculated for each cell and tracked as a contributing component to total evapotranspiration at each simulated daily time step.

Surface evaporation is calculated using a US Class A Pan Evaporative approach derived by Linacre [32]. This approach requires dew point, an environmental metric often not measured at weather stations. To overcome that data limitation, Eccel’s observation that “when the daily minimum water vapor is saturated” the minimum air temperature “reaches dew point”, meaning that daily minimum temperature can be used as a reasonable surrogate for dew point [33].

Surface evaporation is calculated as:

Eq1 EV=(0.015+0.00042∗T+0.000001∗Z)∗(0.8∗I−40+2.5∗F∗Ws∗(T−Td))

where *EV* is the surface evaporation (mm), *T* is temperature (°C), *Z* is elevation (m), *I* is irradiance (W/m^2^), *F* is change in air density by elevation (m), *Ws* is wind speed (m/sec), and *Td* is minimum temperature (°C), the surrogate for dew point. The variable *F* is calculated as:

Eq2 F=1.0−0.000087∗Z

where *Z* is elevation (m).

**Fig 7.**
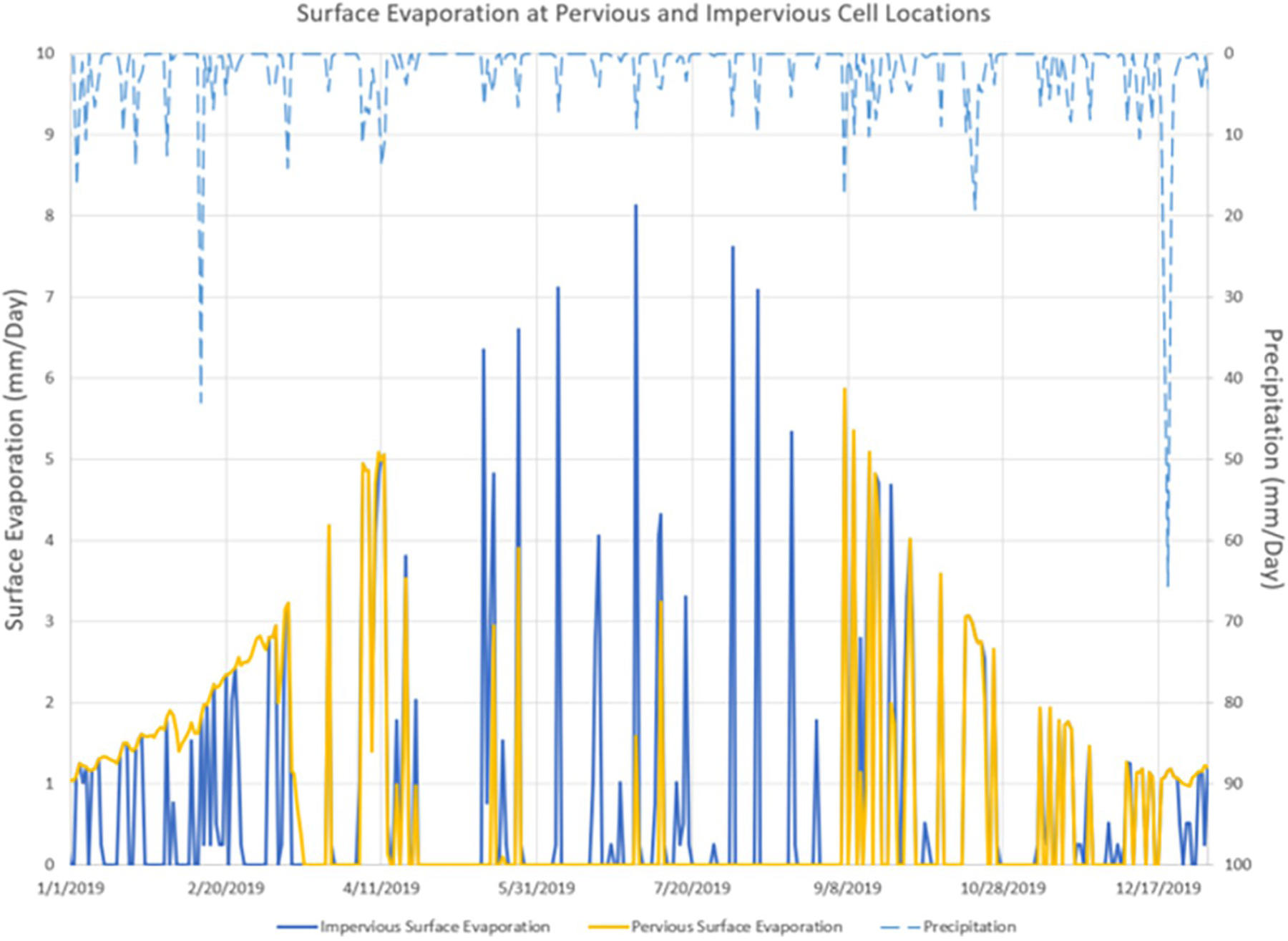
Surface evaporation at a pervious versus impervious cell location for year 2019.

Surface evaporation from two locations were compared to demonstrate the difference loss to the atmosphere due to the inclusion of the *SurfaceEvaporation* model (Fig 7). The evaporation at both the pervious and impervious cell locations start as similar quantities during the first three months of the year (winter season), though at low levels due to the colder weather and as jagged behavior for the impervious location. Surface evaporation increases as the air temperatures increase throughout the spring season. As rainfall events become less frequent and air temperatures increase into the summer season, the pervious cell undergoes less surface evaporation, while the impervious cells surface evaporation quantity increases to as high as 8 mm/d (Fig 7).

### 2.3. VELMA coverages: Urban and natural

**Fig 8.**
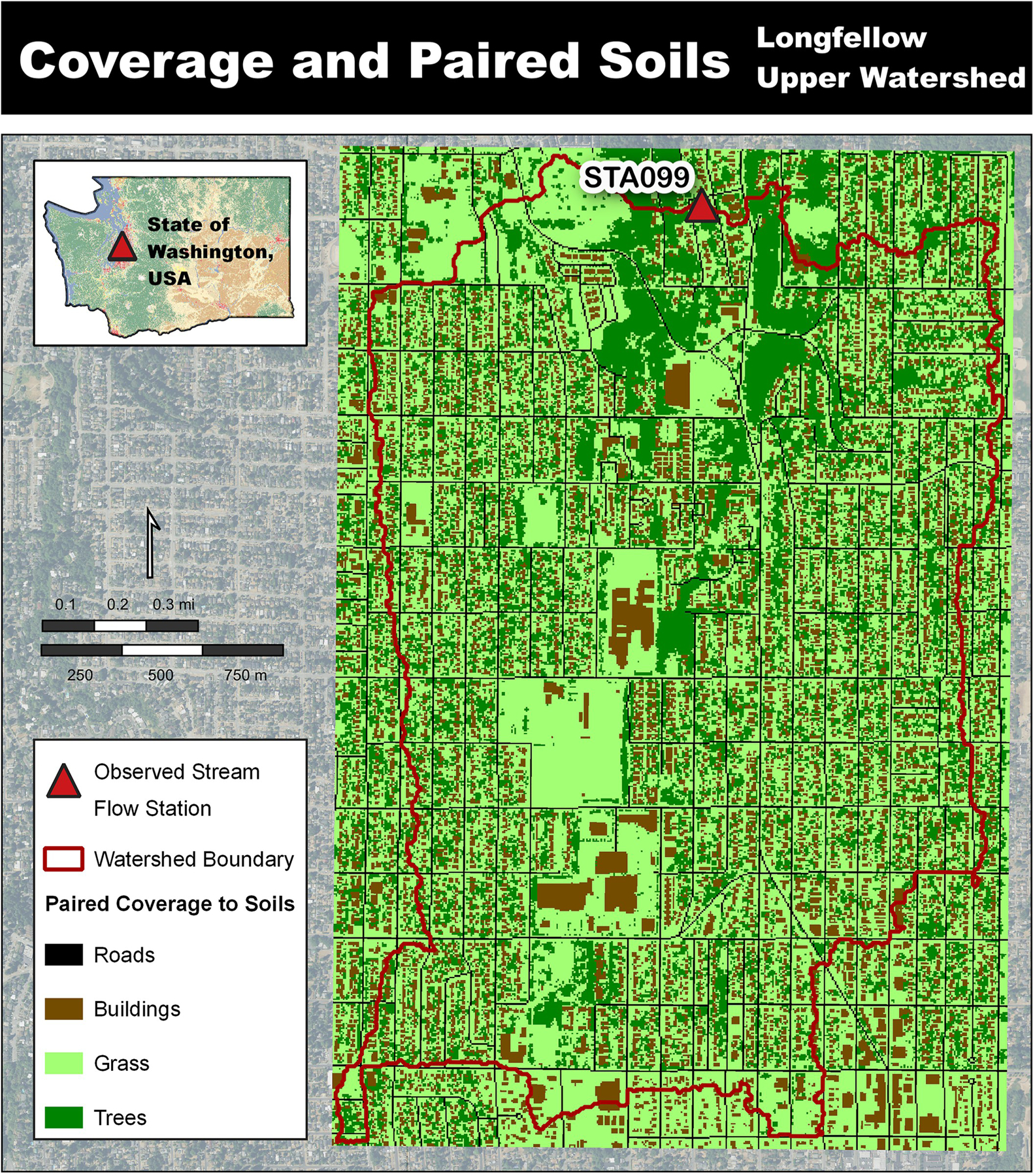
VELMA coverage types with paired soil types [23, 24].

The coverage types for buildings and roads were developed for VELMA urban modeling simulations. These coverage types have exceedingly low biomass levels, PSM parameter values to reflect slowed vegetative recovery and spatially correspond to the higher impervious surface cell locations. The incorporation of a vegetation cover type to primarily impervious grid cells is a necessary VELMA setup development for urban simulations but is not a model framework improvement, though for lucidity of the simulation setups, the landscape cover types are described below (Fig 8). Methods for setting up vegetation cover types are described by McKane et al. [21, 22].

### 2.4. Evaluating VELMA: Current versus improved VELMA

To evaluate how each of the described VELMA enhancements contributed to the realism of simulated Longfellow Creek watershed results, a calibrated VELMA baseline scenario with all four urban modeling enhancements served as the control simulation. Four additional scenarios were then developed, such that each scenario was used to simulate the effect of removing one of the enhancements for comparison to the baseline scenario. This analysis also included a fifth scenario in which all four enhancements were removed. All scenario simulations were conducted using the baseline simulation set of VELMA parameter values (no recalibration across scenarios).

To demonstrate the inclusion and importance of proper water routing within an urban landscape model, we evaluate here the Longfellow Creek watershed observed flow station STA099 versus VELMA simulated flow results under five scenarios. All the following simulations include VELMA’s core dynamic processes affecting soil matrix hydrologic connectivity and vegetation ET. The “baseline” VELMA simulation includes all four new urban model enhancements described above. Each subsequent scenario has one of the urban enhancements removed with other three enhancements left in place to estimate, in comparison to the baseline scenario, how the exclusion of each enhancement limits VELMA’s ability to simulate urban landscape hydrology:

1–4. Baseline: full setup for explicit modeling of water routing through an urban watershed. Simulation includes VELMA core hydrologic processes and urban enhancements 1 through 4 (S1 Data).

1. Impervious Surface: This scenario removed *Enhancement-1* impervious surface, allowing 100% water infiltration into the soil matrix layer one. The curbs and stormwater representation were still in place, therefore excess surface layer water could route out of the watershed through those *Water-Disturbance* mechanisms. Any previously impervious cell containing excess surface water would now have an increased likelihood of transferring water vertically down into the soil matrix, instead of horizontally across the surface layer (S1 Data).
2. No Curbs and MS4 Stormwater System: This scenario removed *Enhancement-2* and *Enhancement-3*, piped networks and curbed streets, respectively. This scenario’s complete lack of curbs and MS4 increases lateral runoff from roads to roadside areas, potentially increasing infiltration in roadside soils, runoff in ditches, and flooding in low lying neighborhoods (S1 Data).
3. Roof-to-Sanitary CSS: This scenario removed *Enhancement-3*, roof-to-CSS piping. For buildings equipped with such piping, roof runoff is transferred to the wastewater treatment plant (WWTP) outside the simulation’s delineation. This scenario allows precipitation falling upon such roofs to drain into a building’s surrounding soil matrix and/or to impervious surfaces (S1 Data).
4. Surface Evaporation: This scenario removed *Enhancement-4*, spatially explicit evaporation from impervious surfaces (S1 Data).
5. All Urban Enhancements: This scenario removed all *Enhancements 1* through *4* in aggregate (i.e., no Curbs, no Inlets to Outlets, no roof-to-CSS drainage, no impervious surface, and no impervious surface evaporation) (S1 Data).

## 3. Results

The VELMA baseline simulation implemented all urban enhancements to more adequately model local, spatially explicit components of the Longfellow Creek urban watershed. In addition to VELMA core ecohydrological processes, the added urban enhancements of impervious surfaces, street curbs, stormwater system inlets to outlets, roof-to-CSS drainage, and surface evaporation are represented in the baseline simulation. The annual hydrology performance varied from satisfactory to excellent for the four calendar years a complete hydrology record was available (Table 3).

Observed Longfellow Creek stream runoff records were complete for the years 2019 and 2022. All years performed well based on the NSE statistics being greater than 0.7 and less than 1.3. The Runoff Fraction (sim/obs) also revealed good simulation performance with the runoff being off by no more than −31% to +17% with 2021 performance of 0.99 being nearly perfect. For baseline model performance and evaluation of modeled urban enhancement through distinct removal, we focused on the results for year 2020 due to the satisfactory Nash-Sutcliffe Efficiency (NSE) goodness-of-fit (0.82) and Runoff Fraction of 0.92 (simulated/observed) statistics.

**Fig 9.**
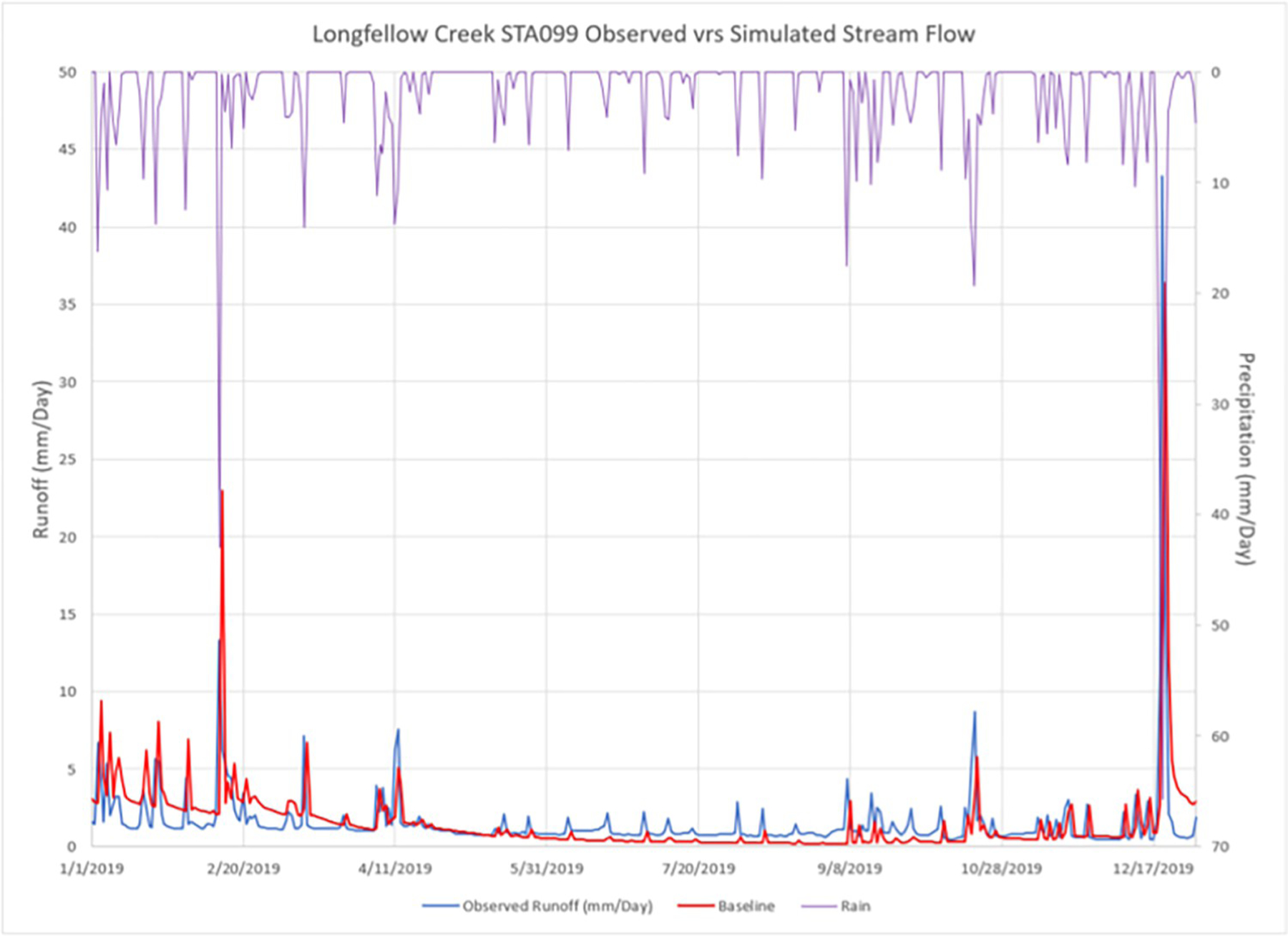
2020 observed versus simulated stream runoff (mm/day, left y axis) at Longfellow Creek gauge station STA099. Precipitation events are displayed upside down (right y axis).

The daily simulated hydrology does have seasonal inconsistencies compared to the observed record. Though the VELMA baseline simulation tended to overestimate fall/winter/spring wet season runoff and underestimate runoff in dry summer months, overall agreement between simulated and observed daily and annual runoff was very good (NSE = 0.82, Table 4 and Fig 9). The following scenario testing focused on 2020 hydrograph (Fig 9). Hydrographs for years 2019, 2021, and 2022 are provided as S1–S3 Figs.

### 3.1. Urban enhancements scenario testing

The VELMA baseline scenario results for Longfellow Creek (Fig 9) were used as a reference against which to compare changes in runoff for the scenarios representing the independent removal of each VELMA urban modeling enhancement. To ensure comparability of runoff results across scenarios, all simulations were conducted using the same timeframe, climate data, and VELMA calibration parameter values.

#### Scenario A: Baseline.

The calibrated Baseline (A) scenario containing all urban enhancements resulted in an excellent goodness-of-fit based on both the NSE and annual runoff fraction (sim/obs). Each subsequent simulation (B through F) experienced a shift in overall annual performance. The scenarios for No Impervious Surface (B), No Roof to CSS (D), and No Surface Evaporation (E) all resulted in increased annual flow compared to the Baseline. In contrast, the No Curbs and MS4 (C), and All Urban Removed (F) scenarios both resulted in decreased annual flow compared to the Baseline scenario (A). Table 4 quantitatively summarizes these scenario differences. Daily flow results per simulation were graphed together to show the seasonal pattern differences among the simulations, provided as S4 Fig.

#### Scenario B: No impervious surface.

**Fig 10.**
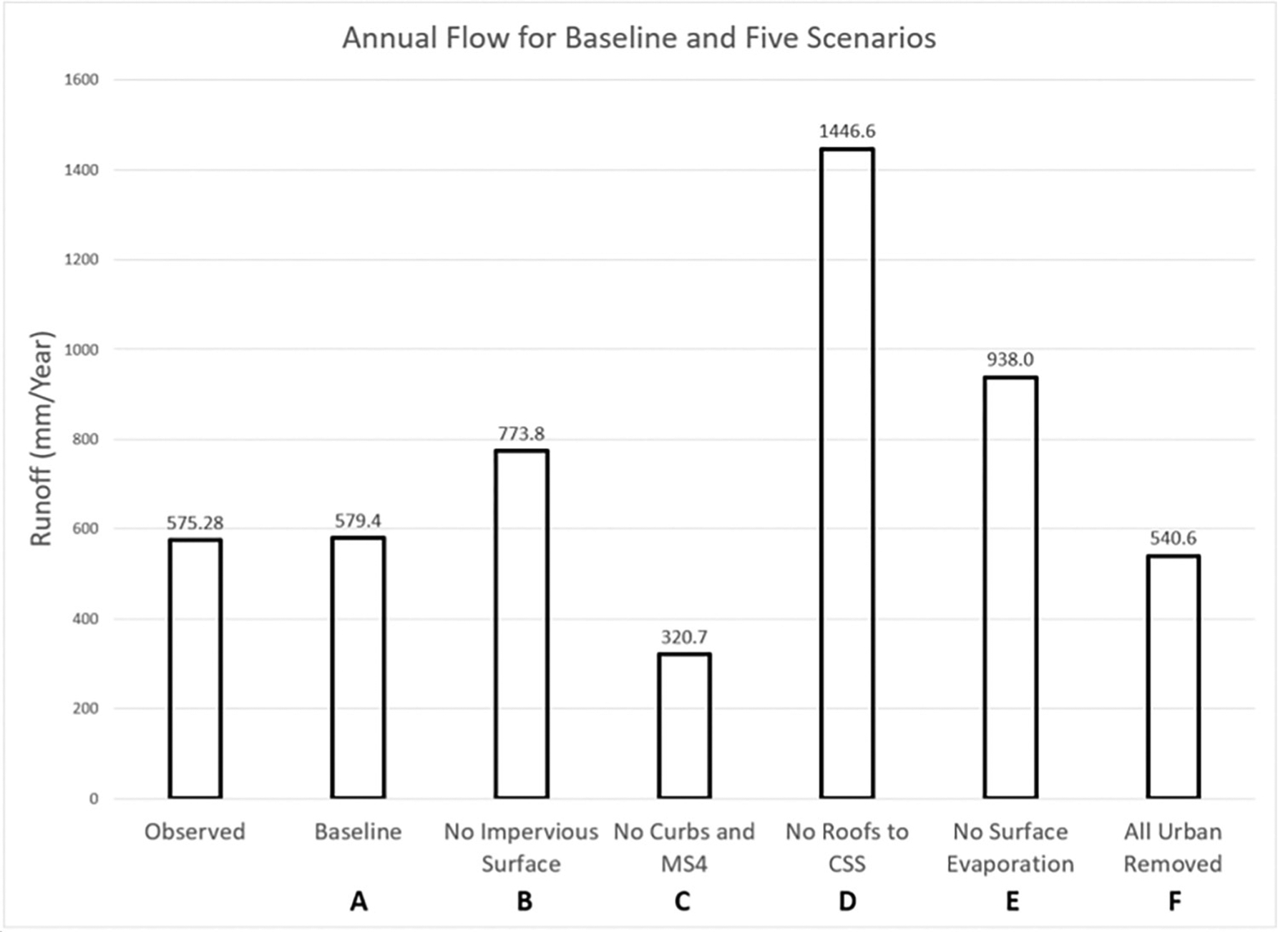
Longfellow Creek 2020 annual total runoff scenarios comparing observed and Baseline scenario results versus each scenario with an urban enhancement removed. Scenarios B through E simulate the removal of one urban modeling enhancement from VELMA. Scenario F Simulates the combined removal of all (A-E) urban enhancements.

The No Impervious Surface scenario (Fig 10B) returned any VELMA enhancements to the VELMA 2.0 default state whereby all grid cell surfaces were 100% permeable. As a result, any previously impervious cell containing surface water would instead allow infiltration of water into to the upper most soil layer until any unsaturated pore space became filled (possible during major storm events but otherwise unlikely). Under this scenario, the curbs and MS4 stormwater representations were still in place; therefore, any water remaining on the surface could route to a storm drain and pipe outfall to the stream. The lack of impervious representation also resulted in increased soil saturation levels and subsurface flow, thereby contributing to a ~1.27x baseline increase in streamflow (Table 4 and Fig 10B). A counteracting factor that would tend to reduce higher subsurface flow and stream runoff are higher rates of evaporation from cells having vegetation cover (grass and trees) compared to their formerly impervious condition (Fig 8). A more detailed analysis of the water budget at fine and coarse watershed scales is needed to resolve which factors most affected the simulated increase in annual runoff for the No Impervious scenario.

#### Scenario C: No curbs and MS4.

The No Curbs and MS4 scenario represents a complete lack of curbed roads and MS4 stormwater system representation. The lack of capacity to transfer water through the stormwater system and into the stream caused a reduction in stream flow, especially during the wetter seasons when surface water is often in excess. The lack of MS4 water inputs directly to the stream caused a 0.56x Baseline overall decrease in annual run-off (Table 4 and Fig 10C). This result was due mainly to surface water being forced to route laterally along impervious surface cells until reaching a cell with an unsaturated pervious soil surface that could retain the water. This was the only scenario to result in a substantial decrease in annual runoff.

#### Scenario D: No Roofs-to-CSS piping.

Some Longfellow Creek watershed roofs have corresponding “lateral drainage pipes” that route water outside the watershed via CSS pipes whose destination is a remote WWTP. The No Roofs-to-CSS Piping scenario simulates the absence of this roof-to-CSS linkage, such that any precipitation falling upon these roofs instead drains to cells adjacent to the building supporting the roof. The net effect was a large 2.14x Baseline scenario increase in annual runoff to Longfellow Creek (Table 4 and Fig 10D). This revealed that with roof-to-CSS a significant portion of the annual rainfall falling within the watershed boundary is routed outside the watershed. It follows that Longfellow Creek annual flow would be that much higher if roof runoff were not being externally rerouted to the extent that it is.

#### Scenario E: No surface evaporation.

The No Surface Evaporation scenario removed the inclusion of the *surfaceEvaporation* model from the simulation. This eliminated evaporative water loss from standing water on VELMA’s surface layer, mostly from impervious surfaces, due to infiltration into pervious soils preventing buildup of surface water except during major storm events. The removal of this urban enhancement increased total watershed annual runoff ~1.39x Baseline scenario (Table 4 and Fig 10E). Though surface evaporation is a relatively small portion of the watershed’s overall water budget, the elimination of evaporative losses from impervious surfaces forces that water to route through other transport mechanisms. For example, lateral surface transport to nearby soils supporting infiltration, higher ET and subsurface flow and stream baseflow, or transport to stormwater MS4 piped flow to the stream.

#### Scenario F: All urban enhancements removed.

This scenario removed all four urban enhancements in combination: No Impervious Surfaces; No Curbs and MS4; No Roofs-to-CSS; and No Surface Evaporation. The overall annual effect suggests these urban enhancements had little impact relative to the Baseline simulation: annual runoff ~1.00x Baseline scenario (Table 4 and Fig 10F). However, as described above, the results for the removal of each individual urban enhancement reveals otherwise (Table 4 and Fig 10). Also, the combined net effect of removing all enhancements decreased the modeled vs observed stream runoff goodness-of-fit Nash-Sutcliffe-Efficiency (NSE) statistic from 0.82 to 0.55. An NSE value of 1.0 is a perfect fit; 0.82 is excellent; 0.55 is a poor-quality result.

Part of the problem is that annual runoff relative to the Baseline scenario decreased for one enhancement removal scenarios while increasing for the other three scenarios. Runoff increases far outweighed decreases, despite the near parity with Baseline runoff when all removal scenarios were simulated together. This suggests that there were interactive effects when all removal scenarios were simulated in combination. A more detailed set of factorial removal simulations is beyond the scope of the present analysis but could potentially help understand underlying process-level interactions for this phenomenon. In any case, this study presents a cautionary result for attempts to model urban landscapes.

## Discussion

The modeling research described here has sought to accurately model ecohydrological processes for a complex urbanized watershed, the Longfellow Creek upper watershed in West Seattle, specifically to identify multi-scale controls (5-m grid cells to whole watershed) on stream runoff imposed by natural and engineered (green and gray) infrastructure. Toward this goal, we applied available high-resolution data describing land-use, imperviousness, and stormwater infrastructure to VELMA’s framework, a spatially explicit ecohydrological model linking hydrological and biogeochemical processes within watersheds. We enhanced VELMA’s capabilities for this project to better represent urban green and gray runoff management systems used to redirect, store, and otherwise control urban runoff for flood control and other human purposes–purposes often accompanied by unintended consequences for stream habitat quality and aquatic life.

These urban modeling enhancements specifically included the kinds, amounts, and locations of 1) impervious surfaces, 2) piped MS4 and CSS stormwater networks, 3) curbed streets, and 4) surface layer evaporation. Applied in unison for VELMA’s Baseline Longfellow Creek watershed scenario, these urban enhancements provided excellent daily and annual runoff performance for stream gauge station STA099 located at the upper watershed pour point (Baseline scenario/observed annual flow = 0.92; NSE goodness-of-fit = 0.82; Table 4).

Four additional VELMA simulation scenarios, each of which removed one or all four urban modeling enhancements, served to isolate each enhancement’s individual and collective impact on stream runoff. The enhancement removal scenario resulted in some cases fitting anticipated responses but others revealing some surprising outcomes.

As anticipated, the No Curbs and MS4 scenario produced much less annual stream runoff (0.56x observed, Table 4), as VELMA gravitationally redirected about half of precipitation falling on now non-curb streets to roadside ditches, lawns, etc., rather than to MS4 drains and pipes due to curbs functioning to keep surface runoff on roads against lateral gravitational flow paths.

Most surprising was the more than doubled annual stream runoff for the No Roofs-to-CSS scenario (2.14x Baseline; Table 4 and Fig 10). This simulation included only those roofs that Seattle Public Utilities GIS data indicated as having roof drains connected to the CSS. The CSS routes that portion of watershed total roof runoff to a WWTP and thence to Puget Sound, thereby bypassing surface and subsurface routing to Longfellow Creek. The amount of annual roof runoff sent to the WWTP is greater than the Baseline scenario annual runoff to Longfellow Creek. This model-based outcome provides insight into a process that would otherwise be difficult to quantify empirically, given that the quantity of CSS water exports from the Longfellow Creek watershed to the WWTP reflects the combined contributions of sanitary and stormwater to total CSS flow.

From a modeling standpoint, had the roof-to-CSS effect not been accounted for during our Baseline calibration, it would not have been possible to develop an accurate VELMA All Urban Enhancements calibration that made ecohydrological sense; VELMA rates of evapotranspiration would have needed to be set unrealistically high (e.g., compared to Sanford and Selnick) through over calibration of coverage parameters to achieve a close match of modeled and observed runoff (Fig 9) [30].

VELMA does not yet simulate storm-driven combined sewer overflows (CSOs). Adding that capability is a top priority for informing CSO remediation planning by communities. With the potential added enhancement for addressing CSOs, VELMA could be used to help identify such solutions, for example, replacement of roof-to-CSS plumbing with roof-to-bioswale plumbing that would filter roof runoff through the soil matrix–an action that could significantly reduce roof runoff contributions to CSOs while also slowing and purifying subsurface runoff (baseflow) to Longfellow Creek–a win-win solution for people and fish.

Also surprising was the simulated increase in annual stream runoff for the No impervious surfaces scenario (1.34x Baseline; Table 4 and Fig 10). Based on long-established observations describing increased peak flow flashiness with increased urbanization, we expected a larger increase in runoff when the large area of impervious surfaces within the watershed was converted to maximum perviousness for this scenario (i.e., changed from 0.0 to 1.0). A major reason for this response is the large amount of roof runoff routed to the CSS and WWTP, so this result is not as surprising as first thought.

Lastly, the No Urban Enhancements scenario also held surprises. For this, VELMA simulated the combined interactive effects of the No Curbs and MS4, No Roofs-to-CSS, No Impervious Surfaces, and No Surface Evaporation scenarios. Though the No Urban Enhancements scenario annual flow closely matched the Baseline (All Urban Enhancements) scenario, the No Urban Enhancements scenario did so by over- or under-estimating observed daily runoff at different times of year, such that its largest daily errors nearly balanced each other (Fig 10). This is a case of arriving at a correct answer for the wrong reasons due to the over- and under-estimating of daily flows resulting in a good overall annual statistical fit. Those reasons would be difficult to pinpoint without a factorial analysis of how the elimination of different combinations of urban enhancements affected the net result described in Fig 10 for this scenario.

## Conclusions

This study demonstrates VELMA’s ability to represent and distinguish the effects of specific engineered and natural features of urban watersheds on the transfer and fate of precipitation inputs across multiple scales–from 5-m grid cells to whole watershed. The novelty here is the added capability to simulate urban infrastructure within an ecohydrology model that already simulated dynamic vegetation growth and disturbance along with dynamic fate and transport of water and nutrients upon the surface (including evapotranspiration) and through the soil matrix. The modeling exercises reported here were designed to assess and demonstrate VELMA’s potential for establishing virtual urban watersheds having these capabilities. This opens new opportunities to help inform stormwater management planning for a variety of purposes.

One such purpose is to provide a multi-scale, process-based hydrological foundation for identifying green and gray infrastructure best practices to improve remediation of contaminant fate and transport in stormwater impacting aquatic life in receiving waters. Within Longfellow Creek coho salmon have been experiencing high rates of prespawn mortality for at least the past two decades [34]. Indeed, this phenomenon has been a primary motivation for this research and is the topic of a companion study by our team and community, tribal, state, and federal partners.

## Supplementary Material

Supplement1 **S1 Fig. 2019 observed versus simulated stream runoff (mm/day, left y axis) at Longfellow Creek gauge station STA099.** Precipitation events are displayed upside down (right y axis). (TIF)
**S2 Fig. 2021 observed versus simulated stream runoff (mm/day, left y axis) at Longfellow Creek gauge station STA099.** Precipitation events are displayed upside down (right y axis). (TIF)
**S3 Fig. 2022 observed versus simulated stream runoff (mm/day, left y axis) at Longfellow Creek gauge station STA099.** Precipitation events are displayed upside down (right y axis). (TIF)
**S4 Fig. Hydrograph of baseline daily runoff compared to all other simulations.** (TIF)
**S1 Data. Zipped folder containing the baseline simulation and subsequent five scenarios.** (ZIP)

## Figures and Tables

**Table 1.** VELMA relevant soil parameters.

VELMA Soil Parameter Name	Description
*surfaceKs*	Surface saturated hydraulic conductivity (mm/day)
*soilColumnDepth*	Total depth divided among layers 1, 2, 3, 4 (mm)
*ksVerticalExponentialDecayFactor*	Multiplier dictating rate change of vertical flow with depth
*ksLateralExponentialDecayFactor*	Multiplier dictating rate change of horizontal flow with depth

**Table 2.** Example Curb-to-Curb, Curb-to-Catchment, Catchment-to-Outflow water disturbances.

# in Map	Disturbance Action	Catchment i-index	Percentage of Volume	Outflow i-index
1	Curb-to-Curb	35764	1.0	35763
2	Curb-to-Curb	35763	1.0	35762
3	Curb-to-Catchment	35762	1.0	35343
4	Catchment-to-Outflow	35343	1.0	35760

**Table 3.** VELMA hydrologic performance for simulated vs. observed runoff at stream gauge station STA099.

Year	2019	2020	2021	2022
Precipitation (mm)	732.1	907.7	957.5	1031.4
Runoff NSE	0.82	0.82	0.72	0.70
Observed Runoff (mm)	575.2	640.4	695.7	670.3
Simulated Runoff (mm)	453.3	589.9	691.8	782.9
Difference (sim-obs)	−121.9	−50.5	−3.9	112.6
Runoff Fraction (sim/obs)	0.79	0.92	0.99	1.17
Total AET (mm)	361.7	376.3	375.3	383.6

**Table 4.** VELMA scenario summary of simulation results.

Scenario	Simulated Result	Runoff NSE (sim vs obs)	Annual Runoff Ratio (sim/obs)
(A) Baseline All Urban Enhancements	Good match to daily and annual observed runoff (Table 3)	0.82 (Table 3)	0.92 (Table 3)
(B) No Impervious surfaces	Annual runoff ~1.34x baseline	0.48	1.27
(C) No Curbs and MS4	Annual runoff ~0.56x baseline	0.52	0.61
(D) No Roofs-to-CSS	Annual runoff ~2.14x baseline	−4.03	2.33
(E) No Surface Evaporation	Annual runoff ~1.39x baseline	0.68	1.51
(F) No Urban Enhancements	Annual runoff ~1.00x baseline (compensatory effects)	0.55	0.92

## Data Availability

All data can be found in the manuscript and supporting information files.
