# Supplementary figures and images for: Improved urban runoff prediction using high-resolution land-use, imperviousness, and stormwater infrastructure data applied to a process-based ecohydrological model
Source: PLOS Water. Author manuscript; Available in PMC 2024 Nov 20. (PMC11110540; doi:10.1371/journal.pwat.0000155)

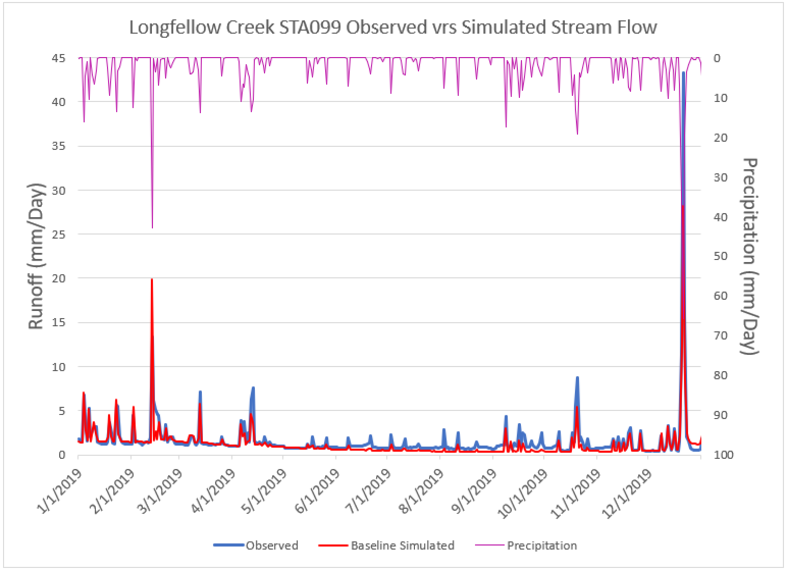

Supplement: Supplement1 — S1 Fig. 2019 observed versus simulated stream runoff (mm/day, left y axis) at Longfellow Creek gauge station STA099. Precipitation events are displayed upside down (right y axis). (TIF) S2 Fig. 2021 observed versus simulated stream runoff (mm/day, left y axis) at Longfellow Creek gauge station STA099. Precipitation events are displayed upside down (right y axis). (TIF) S3 Fig. 2022 observed versus simulated stream runoff (mm/day, left y axis) at Longfellow Creek gauge station STA099. Precipitation events are displayed upside down (right y axis). (TIF) S4 Fig. Hydrograph of baseline daily runoff compared to all other simulations. (TIF) S1 Data. Zipped folder containing the baseline simulation and subsequent five scenarios. (ZIP) [file NIHMS1976877-supplement-Supplement1.zip › pwat.0000155.s001.tif]

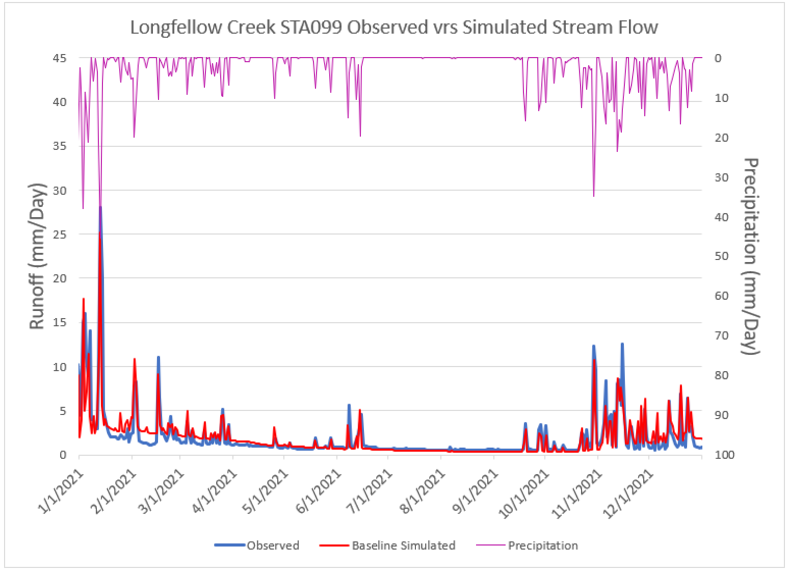

Supplement: Supplement1 — S1 Fig. 2019 observed versus simulated stream runoff (mm/day, left y axis) at Longfellow Creek gauge station STA099. Precipitation events are displayed upside down (right y axis). (TIF) S2 Fig. 2021 observed versus simulated stream runoff (mm/day, left y axis) at Longfellow Creek gauge station STA099. Precipitation events are displayed upside down (right y axis). (TIF) S3 Fig. 2022 observed versus simulated stream runoff (mm/day, left y axis) at Longfellow Creek gauge station STA099. Precipitation events are displayed upside down (right y axis). (TIF) S4 Fig. Hydrograph of baseline daily runoff compared to all other simulations. (TIF) S1 Data. Zipped folder containing the baseline simulation and subsequent five scenarios. (ZIP) [file NIHMS1976877-supplement-Supplement1.zip › pwat.0000155.s002.tif]

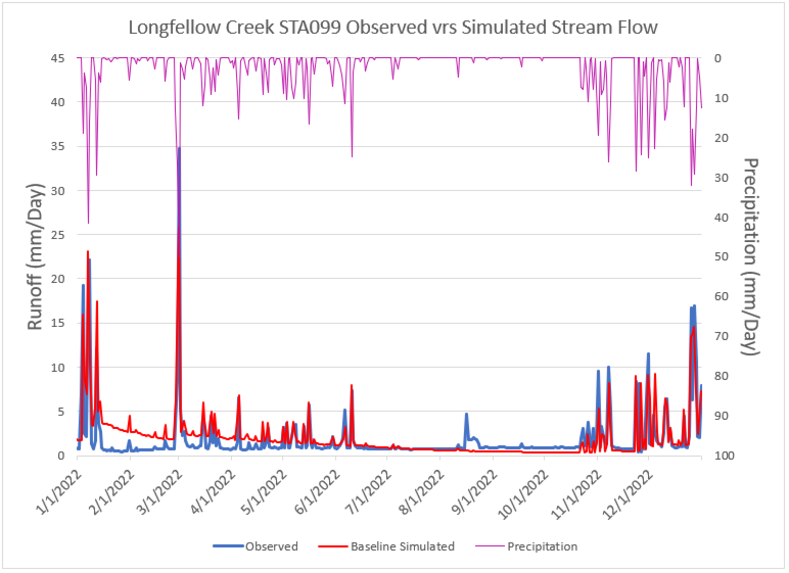

Supplement: Supplement1 — S1 Fig. 2019 observed versus simulated stream runoff (mm/day, left y axis) at Longfellow Creek gauge station STA099. Precipitation events are displayed upside down (right y axis). (TIF) S2 Fig. 2021 observed versus simulated stream runoff (mm/day, left y axis) at Longfellow Creek gauge station STA099. Precipitation events are displayed upside down (right y axis). (TIF) S3 Fig. 2022 observed versus simulated stream runoff (mm/day, left y axis) at Longfellow Creek gauge station STA099. Precipitation events are displayed upside down (right y axis). (TIF) S4 Fig. Hydrograph of baseline daily runoff compared to all other simulations. (TIF) S1 Data. Zipped folder containing the baseline simulation and subsequent five scenarios. (ZIP) [file NIHMS1976877-supplement-Supplement1.zip › pwat.0000155.s003.tif]

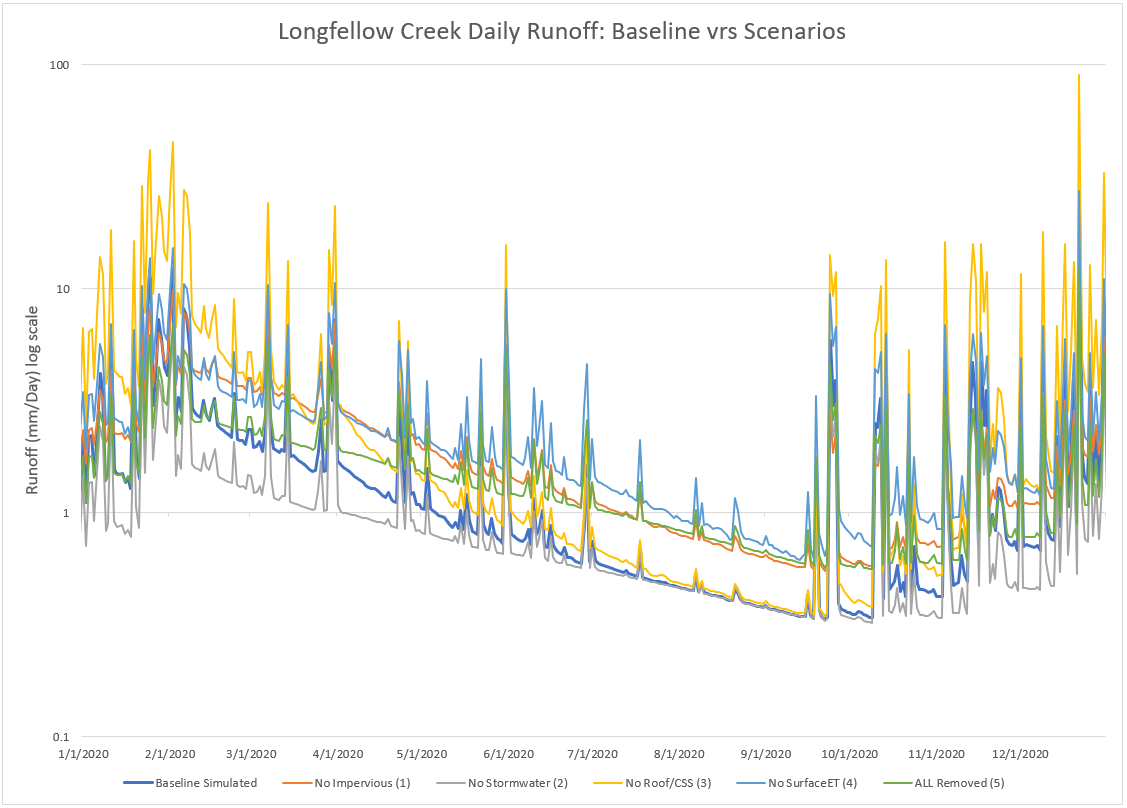

Supplement: Supplement1 — S1 Fig. 2019 observed versus simulated stream runoff (mm/day, left y axis) at Longfellow Creek gauge station STA099. Precipitation events are displayed upside down (right y axis). (TIF) S2 Fig. 2021 observed versus simulated stream runoff (mm/day, left y axis) at Longfellow Creek gauge station STA099. Precipitation events are displayed upside down (right y axis). (TIF) S3 Fig. 2022 observed versus simulated stream runoff (mm/day, left y axis) at Longfellow Creek gauge station STA099. Precipitation events are displayed upside down (right y axis). (TIF) S4 Fig. Hydrograph of baseline daily runoff compared to all other simulations. (TIF) S1 Data. Zipped folder containing the baseline simulation and subsequent five scenarios. (ZIP) [file NIHMS1976877-supplement-Supplement1.zip › pwat.0000155.s004.tif]
